# Probability for surgical treatment in patients with lumbar spinal stenosis according to the stenotic lesion severity: a 5–10-year follow-up study

**DOI:** 10.1186/s12891-022-05510-7

**Published:** 2022-06-14

**Authors:** Dong-Ho Kang, Sanghoon Lee, Ho-Joong Kim, Sang-Min Park, Jin S. Yeom

**Affiliations:** Spine Center and Department of Orthopaedic Surgery, Seoul National University College of Medicine and Seoul National University Bundang Hospital, 166 Gumiro, Bundang-gu, Seongnam, 463-707 Republic of Korea

**Keywords:** Lumbar spinal stenosis, Natural history, Surgical decision, Magnetic resonance imaging, Qualitative grading

## Abstract

**Background:**

We aimed (1) to clarify difference in the natural history of lumbar spinal stenosis (LSS) with respect to surgical treatment according to severity of stenosis on magnetic resonance imaging (MRI) using qualitative grading system and (2) to estimate surgical probabilities depending on radiological severity.

**Methods:**

With the design of retrospective observational study, a total of 1,248 patients diagnosed with LSS between 2011 and 2014 at our hospital were followed up for the mean duration of 7.7 years (5.17–9.8 years). We investigated severity of central and foraminal stenoses on initial MRI using qualitative grading system and whether surgical treatment was performed. Logistic regression models were used to identify risk factors for surgery.

**Results:**

During the mean follow-up period of 7.7 years, grade 3 maximal central stenosis showed the highest percentage of surgical treatment (57.9%–62.3%) with no significant difference in surgical probabilities according to concomitant foraminal stenosis. Surgical probabilities in grade 2 and 3 maximal foraminal stenosis, were 22.2%–62.3% and 33.3%–57.9%, respectively, depending on concomitant central stenosis. Maximal central stenosis of grades 1, 2, and 3 (odds ratio [OR]: 1.79, 2.21, and 6.26, respectively), and maximal foraminal stenosis of grades 2 and 3 (OR: 2.22 and 2.12, respectively) were significant risk factors for surgical treatment.

**Conclusions:**

The high grades of maximal central and foraminal stenoses were risk factors for surgical treatment. Surgical probabilities were 57.9%–62.3% in grade 3 maximal central stenosis, 22.2%–62.3% and 33.3%–57.9%, respectively, in grade 2 and 3 maximal foraminal stenosis during the mean follow-up period of 7.7 years. These results indicate that the natural history of LSS differs according to grade of maximal central and foraminal stenoses.

## Background

Lumbar spinal stenosis (LSS) is the most common disease associated with back pain and walking disability in elderly patients [1, 2]. Previous studies have shown that LSS has a benign clinical course, and conservative treatment including analgesics and steroid injections for symptomatic relief should be considered before surgery [3, 4]. If back pain and walking disability exhibit no improvement despite conservative treatment, surgery is the reasonable option [3]. Surgical decisions are based on clinical symptoms, physical disability, and magnetic resonance imaging (MRI) findings [4–8].

While some studies have reported that the severity of stenosis on MRI does not correspond to the severity of symptoms and has no predictive value for the natural history of LSS [3, 4], other studies have reported that the severity of stenosis is correlated with deterioration of the clinical course [4, 9]. Wessberg et al. observed that patients with dural sac area (DSA) ≥ 0.5 cm^2^ showed spontaneous improvement in the visual analog scale (VAS) score, but those with DSA < 0.5 cm^2^ did not [9]. Herno et al. reported that patients with block stenosis at myelography eventually required surgical decompression [4]. Therefore, consensus is still lacking regarding the probability of surgical decompression according to the severity of stenosis on MRI at diagnosis.

Despite the benign natural history of LSS [4], results of deterioration have been reported in some studies [3, 10]. Due to this uncertainty in the natural history and clinical course, some patients with LSS might continue with ineffective conservative treatment or undergo unnecessary surgery. Therefore, we hypothesized that there would be a difference in the probability of surgical decompression according to the grade of stenosis on MRI. This study aimed (1) to clarify the difference in the natural history of LSS with respect to surgical treatment according to the severity of stenosis on MRI using a qualitative grading system for central and foraminal stenoses and (2) to estimate the probability of surgical treatment depending on the severity of canal stenosis on MRI.

## Methods

### Study design and population

The study was reviewed and approved by the institutional review board of the hospital. This retrospective observational study analyzed the data of patients with LSS through their electronic medical records (EMRs) and picture archiving and communication system (PACS).

Adult patients diagnosed with LSS between 2011 and 2014 at our hospital were included in the study. The diagnosis of LSS was based on radiological evidence of stenotic lesions on lumbar MRI, with corresponding symptoms such as pain, numbness, neurological deficits in the legs and buttocks, neurogenic claudication bladder and bowel dysfunction [11]. The exclusion criteria were death due to life-threatening disease, symptomatic Meyerding grade 3 or higher spondylolisthesis, congenital stenosis, previous spine surgery before initial MRI, spine surgery after initial MRI due to other diseases including herniated vertebral disc, symptomatic Meyerding grade 3 or higher spondylolisthesis, scoliosis, congenital stenosis, vertebral fracture, and malignancy. Patients who did not respond to the telephone interview were also excluded.

**Fig. 1:**
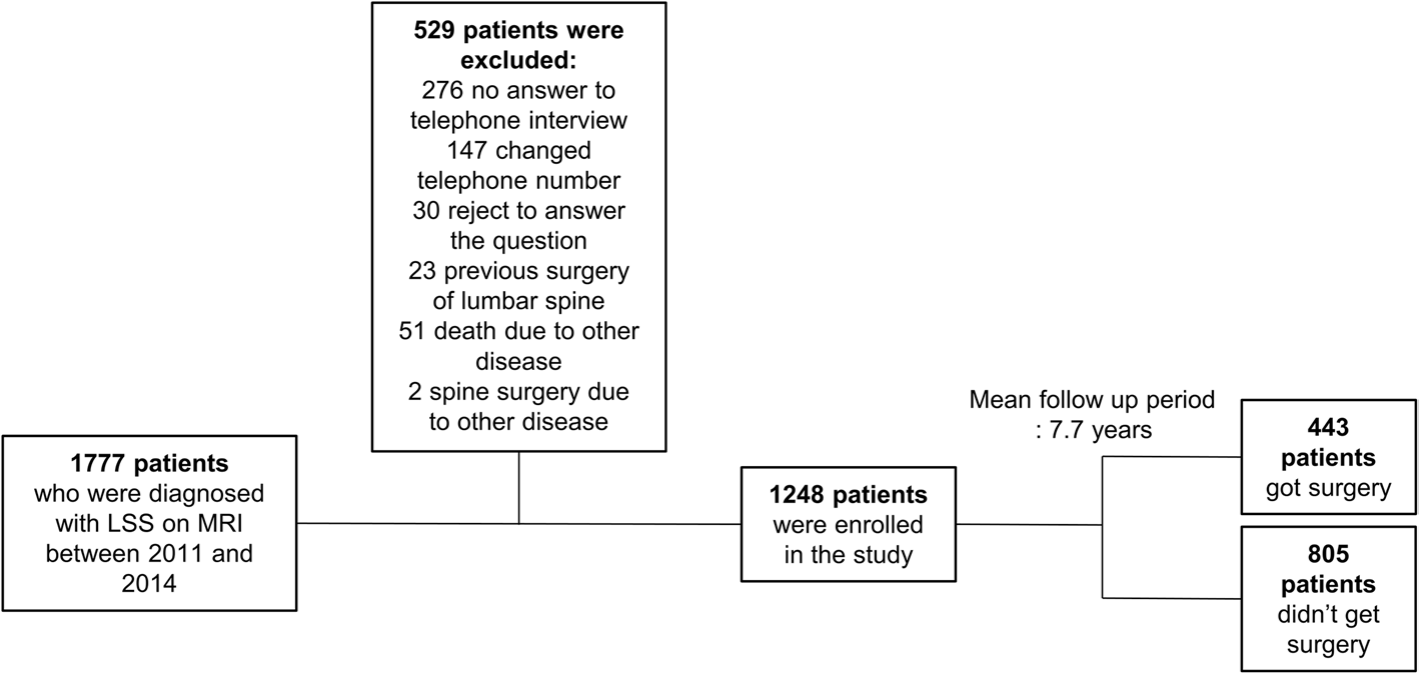
Flowchart of subject recruitment. LSS, lumbar spinal stenosis; MRI, magnetic resonance imaging

All LSS patients were treated surgically under informed consent or preference-based shared decision-making process after sufficient conservative treatment. Surgical treatment was decided in cases with failure of conservative treatment or ongoing neurologic impairment. The EMRs and telephone interviews were reviewed to check whether surgery including posterior decompression, foraminotomy, or fusion surgery was performed for the treatment of LSS, as well as the timing of the operation during a follow-up period of 5.2–9.8 years until 2020. Altogether, 1,777 patients with LSS who underwent MRI were reviewed. After exclusion, 1,248 patients were finally included, with a mean follow-up duration of 7.7 years (Fig. 1).

### Diagnostic imaging

All patients with LSS underwent MRI examination. All images were obtained through electronic access to PACS, which is made up of Digital Imaging and Communications in Medicine format. All axial and sagittal T1- and T2-weighted images of the lumbar spine were reviewed by the radiology department and the two authors. The severity of central and foraminal stenotic lesions was qualitatively graded using T2-weighted axial images at five available disc levels (L1–S1). We classified the severity of stenotic lesions using the Lee classification system to grade the severity of central and foraminal stenotic lesions, which showed excellent inter-reader and intra-reader reliability (Table 1) [12–14]. The narrowest lesions in the central canal and neural foramen which could explain the patient’s symptoms on the initial electric medical records were defined as the maximal central and maximal foraminal stenoses, respectively. We also investigated the number of stenotic levels; thus, the number of disc levels with qualitative grading of the stenotic lesion was not zero.

**Table 1:** The qualitative grading systems of lumbar spinal stenosis on MRI

	Grade 0	Grade 1	Grade 2	Grade 3
Central lesion (Lee et al. 2011) [12]	No stenosis	Mild stenosis with clear separation of each cauda equine	Moderate stenosis with some cauda equina aggregation	Severe stenosis with the entire cauda equina as a bundle
Foraminal lesion (Lee et al. 2010) [14]	Normal	Perineural fat obliteration in the two opposing directions	Perineural fat obliteration in the four directions	Nerve root collapse or morphologic change

### Statistical analysis

Differences in continuous data between the groups were assessed using t test and analysis of variance. Differences in categorical data were assessed using the chi-squared test and linear-by-linear association. The potential risk factors for surgery, such as age, sex, morphologic grade of the maximal central and foraminal stenosis, and the number of central and foraminal stenotic levels, were examined using a logistic regression model. Variables significantly associated with surgical treatment (*p* < 0.20) in the univariate logistic regression analysis were entered into the multivariate logistic regression model, which was used to calculate the odds ratios (OR) and 95% confidence interval (CI) of variables to predict surgical treatment using the backward elimination method. Survival data were analyzed using Kaplan–Meier survival curves and log-rank tests. IBM SPSS statistics version 19.0 (IBM Corp., Armonk, NY, USA) was used for statistical analysis.

## Results

Among the 1,248 patients with LSS with a mean follow-up duration ± standard deviation (SD) of 7.7 ± 1.1 years, 443 (35.5%) patients underwent surgery. The mean age ± SD of the surgical group was 73.6 ± 11.0 years, which was significantly higher than that of the nonsurgical group (mean age ± SD, 68.4 ± 15.3 years) (*p* < 0.001). No significant difference was observed in the sex ratio between the groups (*p* = 0.959).

**Fig. 2:**
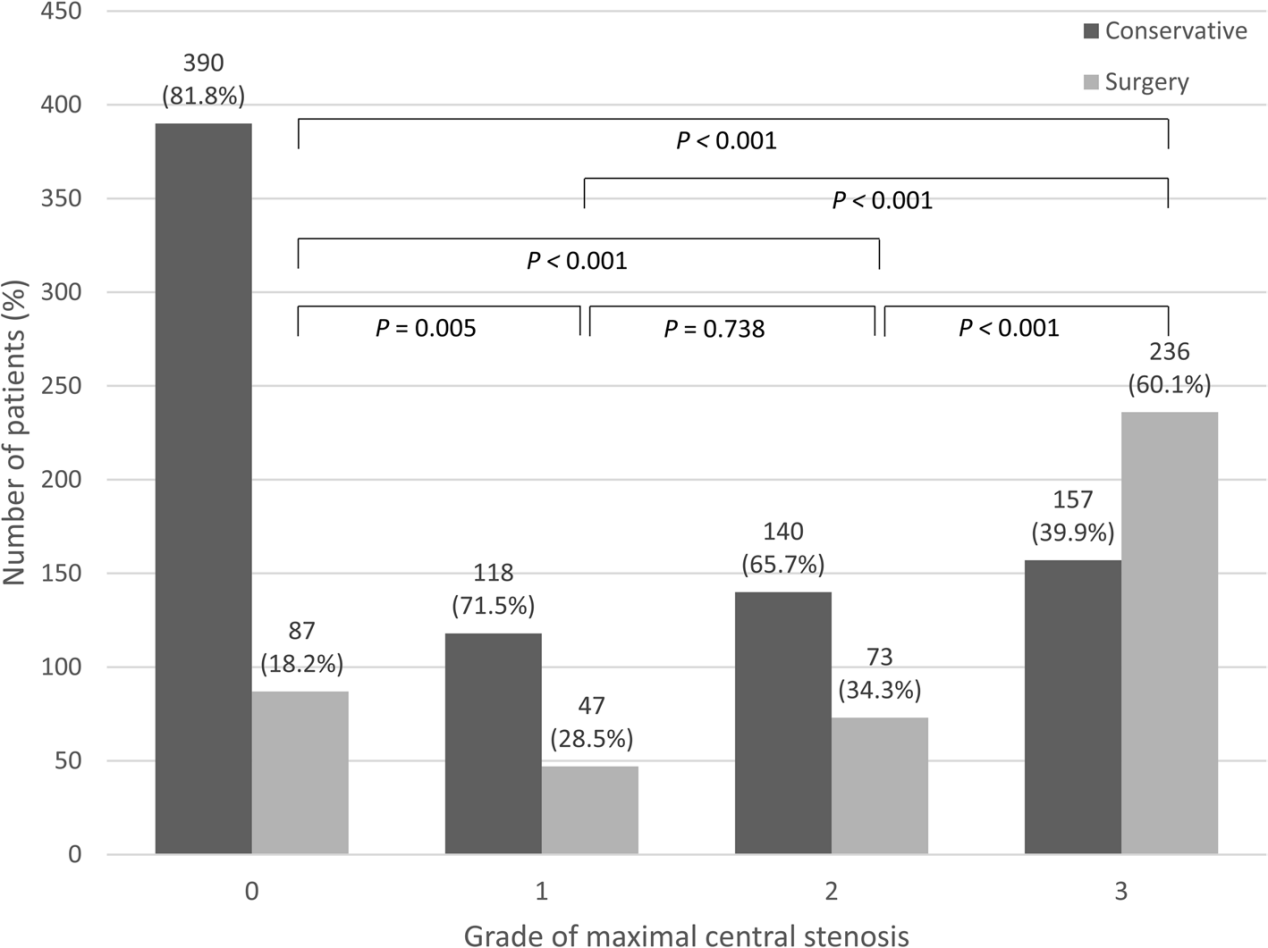
The number of surgical and conservative patients according to the grade of maximal central stenosis on MRI. There was significant difference in the ratio of surgical patients according to the grade of maximal central stenosis except between grades 1 and 2

**Fig. 3:**
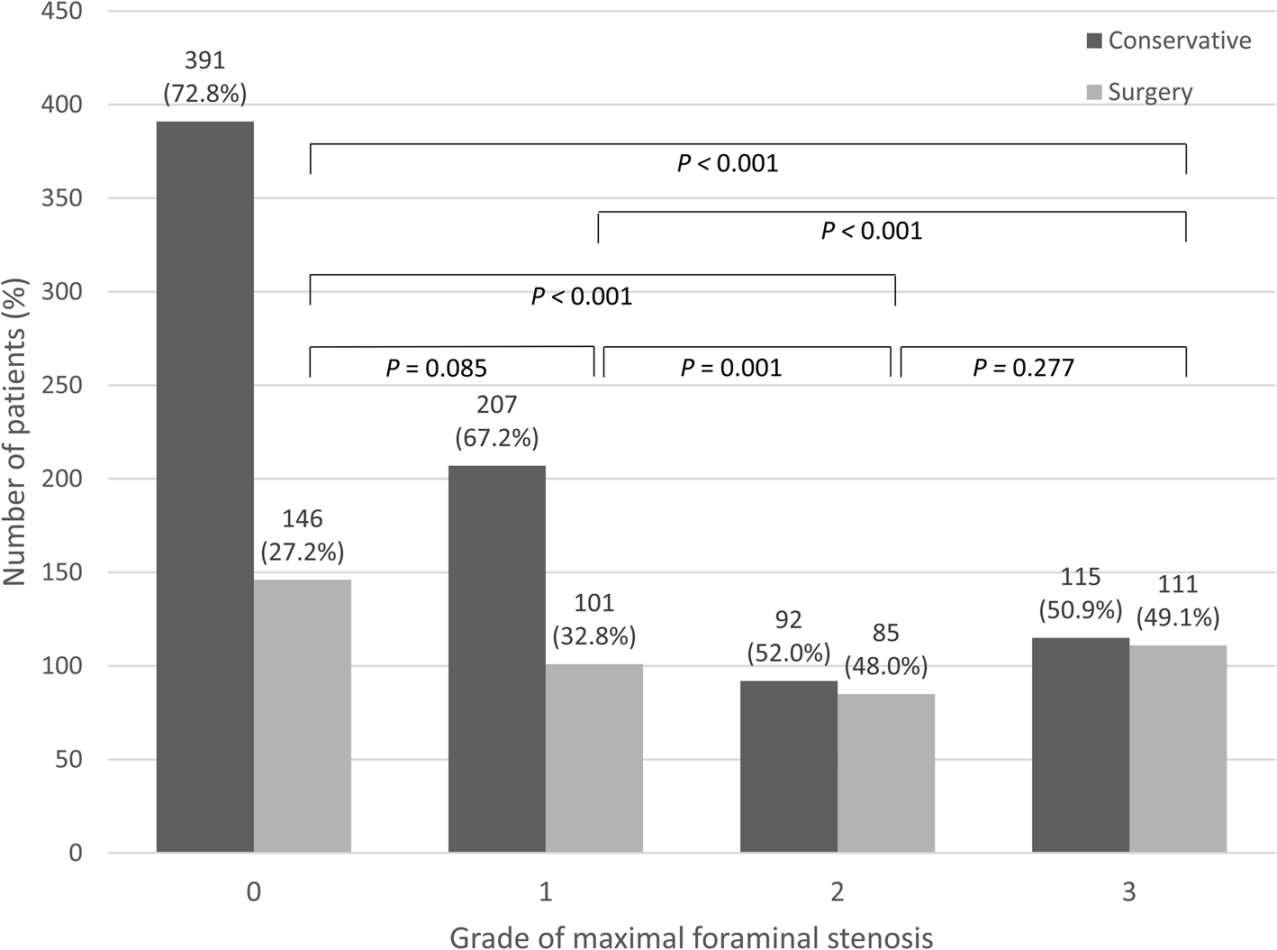
The number of surgical and conservative patients according to the grade of maximal foraminal stenosis on MRI. There was significant difference in the ratio of surgical patients according to the grade of maximal foraminal stenosis except between grades 0 and 1, and between grades 2 and 3

In case of central lesions, the proportion of patients who underwent surgery significantly increased with an increase in the grade of maximal central stenosis (*p* < 0.001), whereas no significant difference was observed between grades 1 and 2 of maximal central stenosis (*p* = 0.738) (Fig. 2). In case of foraminal lesions, the proportion of surgical candidates significantly increased with an increase in the grade of maximal foraminal stenosis (*p* < 0.001), whereas no significant difference was observed between grades 1 and 2 (*p* = 0.085), and between grades 2 and 3 (*p* = 0.277) of maximal foraminal stenosis (Fig. 3).

Surgical probabilities in grade 1, 2 maximal central stenosis were 22.5%–45.0%, 22.2%–41.7%, respectively, according to concomitant grades of maximal foraminal stenosis (Table 2). Grade 3 maximal central stenosis showed the highest percentage of surgical treatment (57.9%–62.3%) with no significant difference in surgical probabilities according to concomitant grades of maximal foraminal stenosis. When there is no concomitant central stenosis (grade 0 maximal central stenosis), the percentage of surgical patients of grade 2 and 3 maximal foraminal stenosis (44.3% and 46.8%, respectively) was significantly higher than that of grade 0 and 1 maximal foraminal stenosis (7.5% and 11.4%, respectively) (*p* < 0.001). The percentage of surgical patients increases significantly from grade 0 to grade 3 concomitant maximal central stenosis in grade 0 (7.5%–61.0%) and 1 (11.4%–59.1%) of maximal foraminal stenosis. Surgical probabilities in grade 2 and 3 maximal foraminal stenosis, were 22.2%–62.3% and 33.3%–57.9%, respectively, according to the grades of concomitant maximal central stenosis.

**Table 2:** Percentage of surgical patients according to combination of grades of maximal central and foraminal stenoses

		Grade of maximal central stenosis				*P* value^a^
		0	1	2	3	
Grade of maximal foraminal stenosis	0	7.5%	22.5%	30.6%	61.0%	< 0.001
	1	11.4%	28.3%	39.6%	59.1%	< 0.001
	2	44.3%	45.0%	22.2%	62.3%	0.084
	3	46.8%	33.3%	41.7%	57.9%	0.122
*P* value^a^		< 0.001	0.110	0.395	0.738	

^a^linear-by-linear association test was used

In a logistic regression, maximal central stenosis of grades 1, 2, and 3 (OR [95% CI]: 1.79 [1.18–2.71], 2.21 [1.52–3.20], and 6.26 [4.59–8.56], respectively), and maximal foraminal stenosis of grades 2 and 3 (OR [95% CI]: 2.22 [1.52–3.24] and 2.12 [1.50–3.00], respectively) were significant risk factors for surgical treatment, whereas other variables including age, sex, and the number of central and foraminal stenotic levels were not significant (Table 3).

**Table 3:** Univariate and multivariate logistic regression analyses of risk factors of surgical treatment

	Univariate analysis		Multivariate analysis	
	Odds ratio (95% CI)	*P* value	Odds ratio (95% CI)	*P* value
Age (years)	1.03(1.02–1.04)	** < 0.001**		0.197
Sex (male)	0.99 (0.78–1.27)	0.959		
Maximum grade of central stenosis		** < 0.001**		** < 0.001**
Grade 1^a^	1.79 (1.19–2.69)	**0.006**	1.79 (1.18–2.71)	**0.006**
Grade 2^a^	2.34 (1.62–3.37)	** < 0.001**	2.21 (1.52–3.20)	** < 0.001**
Grade 3^a^	6.74 (4.95–9.17)	** < 0.001**	6.26 (4.59–8.56)	** < 0.001**
Maximum grade of foraminal stenosis		** < 0.001**		** < 0.001**
Grade 1^a^	1.31 (0.96–1.77)	0.085	1.25 (0.91–1.73)	0.174
Grade 2^a^	2.47 (1.74–3.51)	** < 0.001**	2.22 (1.52–3.24)	** < 0.001**
Grade 3^a^	2.59 (1.87–3.57)	** < 0.001**	2.12 (1.50–3.00)	** < 0.001**
The number of central stenotic levels		** < 0.001**		0.150
1^b^	3.72 (2.72–5.09)	** < 0.001**		0.011
2^b^	3.64 (2.59–5.13)	** < 0.001**		0.228
3^b^	4.40 (2.89–6.70)	** < 0.001**		0.282
4^b^	4.47 (2.34–8.55)	** < 0.001**		0.566
5^b^	2.98 (0.82–10.79)	0.096		0.199
The number of foraminal stenotic levels		** < 0.001**		0.407
1^b^	1.77 (1.34–2.35)	** < 0.001**		0.144
2^b^	1.99 (1.44–2.74)	** < 0.001**		0.374
3^b^	2.40 (1.47–3.94)	**0.001**		0.462
4^b^	3.01 (1.14–7.96)	**0.026**		0.775
5^b^	0.89 (0.09–8.65)	0.922		

^a^Odds compared to grade 0,

^b^Odds compared to 0 level

**Fig. 4:**
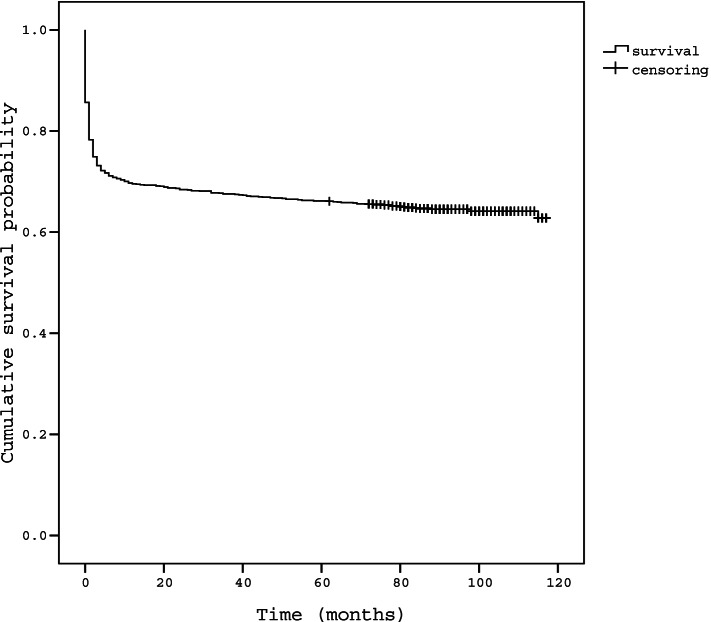
Kaplan–Meier survival curve of overall LSS patients

**Fig. 5:**
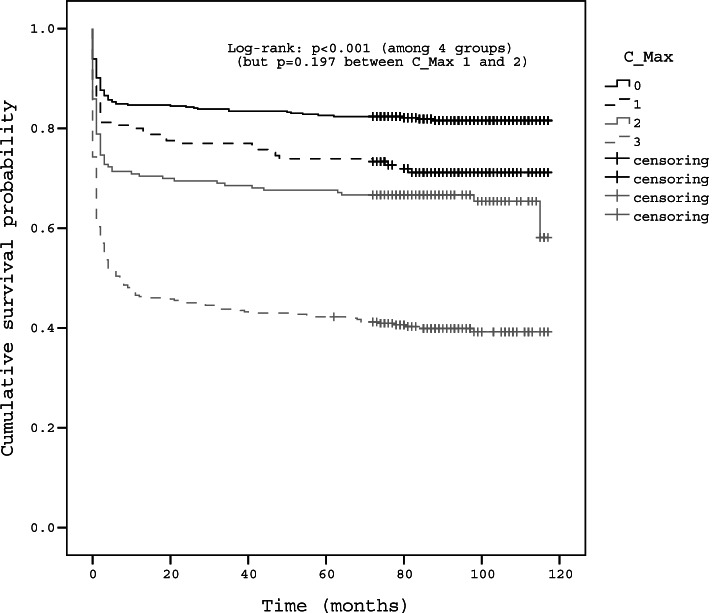
Kaplan–Meier survival curve according to the grade of maximal central stenosis. No significant difference was observed in the survival curve between grades 1 and 2 of maximal central stenosis (*p* = 0.197)

**Fig. 6:**
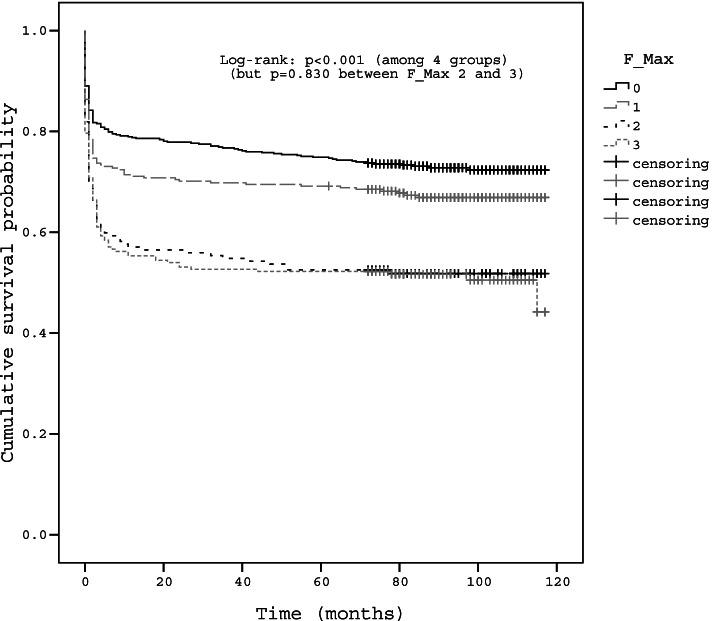
Kaplan–Meier survival curve according to the grade of maximal foraminal stenosis. No significant difference was observed in the survival curve between grades 2 and 3 maximal foraminal stenosis (*p* = 0.830)

Kaplan–Meier curves and log-rank analyses showed significantly different rates of surgical treatment according to the grades of maximal central and foraminal stenoses (Figs. 4, 5, 6). Higher grades were associated with a higher rate of subsequent surgery, but no significant difference was observed in the survival curve between grades 1 and 2 of maximal central stenosis (*p* = 0.197), and between grades 2 and 3 maximal foraminal stenosis (*p* = 0.830). The survival curves showed plateau after initial steep drop for each grade, but survival rate did not actually converge to a constant value and decreases over time (Fig. 4). The survival curve of grade 1 maximal central stenosis approached that of grade 2 maximal central stenosis over time (Fig. 5). The slope of the plateau part of the survival curve is similar among each grade of stenosis (Figs. 5, 6).

## Discussion

The present study showed that the grades of maximal central and foraminal stenoses on initial MRI are risk factors for subsequent surgery in patients with LSS in the course of the disease. During the mean 7.7 years of follow-up period, surgical probabilities in grade 1,2 and 3 maximal central stenosis were 22.5%–45.0%, 22.2%–41.7% and 57.9%–62.3%, respectively, depending on the concomitant grades of maximal foraminal stenosis. Surgical probabilities in grade 2 and 3 maximal foraminal stenosis were 22.2%–62.3% and 33.3%–57.9%, respectively, depending on the concomitant grades of maximal central stenosis. Grade 3 maximal central stenosis showed the highest OR (6.26) for surgical treatment and the highest percentage (57.9%–62.3%). These results imply that the natural history of patients with LSS in the view of the surgical treatment would depend on the grades of maximal central and foraminal stenoses on MRI.

These findings are consistent with those from Schizas’ study, which showed that a greater proportion of patients with severe stenosis based on MRI findings underwent surgery compared to the mild group [15]. Some studies have reported contrasting results, indicating that the severity of stenosis on MRI had no predictive value for the natural history of LSS [3, 4]. However, they used the anterior–posterior diameter of the spinal canal on MRI as a radiological parameter, which was not adequate to accurately assess the degree of neural tissue impingement. Therefore, the morphological classification that reflects neural impingement would be more suitable in both prediction of the disease progress and assess of the severity of stenosis.

A critical point of this study is that we did not access any conservative treatment which patients had taken during the follow-up period. This might be an inherent limitation from retrospective design and long-term follow-up study. However, there has been no study which advocates any conservative can make a change of natural history in LSS. Therefore, this absence of information about conservative treatment would not influence the present conclusion. Likewise, any clinical outcome such as the level of pain and/or disability due to LSS was not assessed during the follow-up period. It might be inappropriate to judge the natural course of LSS using MRI alone, without considering clinical symptoms and other factors, because the surgical decision is made by the complex mechanism both in patients and surgeons. However, it is well-known that the symptoms of LSS fluctuate with the time of its natural course even without change of stenosis [16–18]. Therefore, it might be plausible that the patients who underwent surgical treatment would have progressively increased pain intensity and severe disability in this study and vice versa.

High grades of maximal stenosis (grades 1, 2, and 3 maximal central stenosis and grades 2 and 3 maximal foraminal stenosis) were significant risk factors for surgical treatment. These observations are congruent with previous studies in which LSS patients with severe stenosis on MRI showed no improvement in VAS score during course of disease [9] and patients with block stenosis at myelography eventually needed surgical decompression [4]. Surgical probability in grade 3 maximal central stenosis (57.9%–62.3%) were higher than those in grade 2 and 3 maximal foraminal stenosis (22.2%–62.3% and 33.3%–57.9%, respectively) (Table 2). Grade 3 maximal central stenosis showed the higher OR (6.26) of surgical treatment than grade 2 and 3 maximal foraminal stenosis (2.12 and 2.22, respectively) (Table 3). These findings suggest that surgical probability is more affected by severe central stenosis than by severe foraminal stenosis. There was no significant difference in surgical probabilities between grades 1 and 2 maximal central stenosis regardless of the grade of foraminal stenosis in subgroup analysis (Table 2) and no significant difference in the survival curve between grades 1 and 2 of maximal central stenosis (Fig. 5). The possible explanation for those findings is that clinical symptom or neurological impairment of patients of grade 1 maximal central stenosis might have not differed from grade 2 maximal central stenosis, which is consistent with Andrasinova’s study showing no significant difference in Neurological Impairment Score in LSS between grades B and C of Schizas morphologic classification [5].

Moreover, the finding that the survival curve of grade 1 maximal central stenosis did not converge to a constant value and approached the curve of grade 2 maximal central stenosis also indicates similar rates of surgical treatment between grade 1 and 2 of maximal central stenoses (Fig. 5). The slope of the plateau part of the survival curve is similar among each grade of stenosis, which means that the grade of stenosis on MRI does not affect the symptoms indicating the surgery. The plateau after initial steep drop for each grade in survival curve can be found in previous study. In Amundsen’s partially randomized 10-year follow-up study about natural history of LSS, this plateau could had been observed from that study showing that crossover from conservative to surgical treatment occurred during initial period of 3 to 27 month and treatment result during the final 6 years of the follow-up period were relatively stable [3]. This initial crossover and stable period of final 6 year can explain the initial steep drop and plateau of survival curve in our study, and this imply that the initial response of conservative treatment is important to determine patient's treatment plan. Thus, the initial treatment response could be regarded more important for surgical decision than the later symptom which is represented by the slope of the plateau part of the survival curve similar among each grade of stenosis (Fig. 5, 6). The initial steep drop in the survival curve also would be associated with the place where this study was conducted, which was the tertiary hospital and almost all patients had had adequate conservative treatment before inclusion of the present study. The plateau of the survival curve after initial drop and no intersection of survival curves could means that disease progression of LSS represent generally slow and benign nature. These findings were consistent with previous studies which have advocated the benign nature of LSS progression [3, 4].

The present study has some limitations. Due to the inherent shortcoming of the retrospective study design, we did not assess other factors that might affect the surgical decision, including socioeconomic status, race, ethnicity, and clinical symptoms. However, in the country in which this study was conducted, the research population comprised a single race and a single ethnic group. In addition, all individuals were enrolled in the national medical insurance; thus, the burden of treatment costs would not differ considerably according to the socioeconomic status. Likewise, the clinical symptoms of the included patients might have fluctuated during the long-term follow-up period, with patients with worsening back pain or leg pain undergoing surgical treatment and vice versa. Because the surgical decisions in this study were made under informed consent or preference-based shared decision-making process rather than the surgeon's sole decision, patients who underwent surgical treatment likely had severe and refractory symptoms despite receiving conservative treatments before surgery. Thus, the present results would help physicians to estimate the surgical probability during the follow-up period, based on the stenotic severity on initial MRI.

## Conclusions

In conclusion, this study highlights the difference in the natural history of LSS with respect to surgical treatment depending on the severity of stenosis. Altogether, 57.9%–62.3% of patients with grade 3 maximal central stenosis eventually underwent surgery during the mean 7.7 years of follow-up period. Therefore, the severity of stenosis on MRI at the time of diagnosis can predict the probability of surgical treatment, and the natural history in the view of surgical treatment depends on the grade of stenosis.

## Data Availability

The datasets generated and/or analyzed during the current study are not publicly available due to patient privacy but are available from the corresponding author on reasonable request.

## Abbreviations

LSS: Lumbar spinal stenosis
MRI: Magnetic resonance imaging
OR: Odds ratio
CI: Confidence interval
EMRs: Electronic medical records
PACS: Picture archiving and communication system
SD: Standard deviation
